# Determination of Eight Coccidiostats in Eggs by Liquid–Liquid Extraction–Solid-Phase Extraction and Liquid Chromatography–Tandem Mass Spectrometry

**DOI:** 10.3390/molecules25040987

**Published:** 2020-02-22

**Authors:** Bo Wang, Jianyu Liu, Xia Zhao, Kaizhou Xie, Zhixiang Diao, Genxi Zhang, Tao Zhang, Guojun Dai

**Affiliations:** 1 College of Veterinary Medicine, Yangzhou University, Yangzhou 225009, China; yzwbo168@163.com; 2 Joint International Research Laboratory of Agriculture & Agri-Product Safety, Yangzhou University, Yangzhou 225009, China; 18752540384@139.com (J.L.); 18252711481@139.com (X.Z.); 18352764521@163.com (Z.D.); zgx1588@126.com (G.Z.); zhangt@yzu.edu.cn (T.Z.); gjdai@163.com (G.D.); 3 College of Animal Science and Technology, Yangzhou University, Yangzhou 225009, China

**Keywords:** coccidiostats, eggs, HPLC–MS/MS, UPLC–MS/MS, LLE, SPE

## Abstract

A method for the simultaneous determination of robenidine, halofuginone, lasalocid, monensin, nigericin, salinomycin, narasin, and maduramicin residues in eggs by liquid chromatography–tandem mass spectrometry (LC–MS/MS) was developed. The sample preparation method used a combination of liquid–liquid extraction (LLE) and solid-phase extraction (SPE) technology to extract and purify these target compounds from eggs. The target compounds were separated by gradient elution using high-performance liquid chromatography (HPLC) and ultra-performance liquid chromatography (UPLC). Tandem mass spectrometry was used to quantitatively and qualitatively analyze the target compounds via electrospray ionization (ESI^+^) and multiple reaction monitoring mode. The HPLC–MS/MS and UPLC–MS/MS methods were validated according to the requirements defined by the European Union and the Food and Drug Administration. The limits of detection and limits of quantification of the eight coccidiostats in eggs were 0.23–0.52 µg/kg and 0.82–1.73 µg/kg for HPLC–MS/MS, and 0.16-0.42 µg/kg and 0.81-1.25 µg/kg for UPLC–MS/MS, respectively. The eggs were spiked with four concentrations of the eight coccidiostats, and the HPLC–MS/MS and UPLC–MS/MS average recoveries were all higher than 71.69% and 72.26%, respectively. Compared with the HPLC–MS/MS method, utilizing UPLC–MS/MS had the advantages of low reagent consumption, a short detection time, and high recovery and precision. Finally, the HPLC–MS/MS and UPLC–MS/MS methods were successfully applied to detect eight coccidiostats in 40 eggs.

## 1. Introduction

Coccidiosis is a highly infectious parasitic disease caused by a single-celled organism of the Eimeriidae species. Eimeria is a parasite that is found in the gut of the host and multiplies in the intestinal tract, leading to intestinal tissue damage, reducing the food intake of poultry and the nutrient absorption rate; thus, serious losses are incurred regarding poultry meat and egg production [1,2,3]. Therefore, coccidiosis is one of the most common diseases that causes serious economic losses to farmers of modern, high-density, intensive poultry farms.

To reduce the economic loss caused by coccidiosis, anticoccidial drugs are widely used in the treatment of coccidiosis. Anticoccidial drugs include a wide range of polyether antibiotics (lasalocid, monensin, salinomycin, etc.), synthetic chemical drugs (sulfanilamide groups, halofuginone, robenidine, etc.) and so on. Monensin, salinomycin, and lasalocid have good anticoccidial activity and promote growth function [4]. The mechanism of the anticoccidial activity of antibiotics is different from that of synthetic chemical drugs, so cross-resistance does not occur between the classes, and they can be used interchangeably or in combination to prolong and enhance the ability of disease protection. The European Union [5] has allowed anticoccidial drugs as additives for the production of poultry feed, but use in the production of laying hens is banned. However, anticoccidial residues are often found in eggs, which may be the result of cross-contamination from feed production [6,7,8]. Studies have shown that anticoccidial drugs in feed will be transferred to eggs [9].

To detect anticoccidial residues in eggs, a reliable and sensitive method is required. Many of the methods reported in the past have been used to detect the residues of anticoccidials, such as the commonly used high performance liquid chromatography in series with an ultraviolet (UV) detector [10,11] or a fluorescence detector (FLD) [12]. However, due to the low UV and FLD absorbance of polyether antibiotics, it is often necessary for them to be derivatized for detection, which is not only time-consuming but the sample pretreatment becomes more complex. In addition, various analytical methods have been used to measure anticoccidial drugs in eggs, chicken tissue, milk, duck muscle, pork, beef, and other animal foods, including competitive enzyme-linked immunosorbent assays (cELISA) [13], micellar electrokinetic chromatography (MEKC) [14], flow cytometry-based immunoassays (FCIA) [15], hydrophilic interaction liquid chromatography–tandem mass spectrometry (HILIC–MS/MS) [16], and liquid chromatography–tandem mass spectrometry (LC–MS/MS) [17,18,19,20,21,22,23,24,25,26,27,28,29,30,31,32,33,34,35,36,37,38,39,40,41]. Compared with other detection methods, the LC–MS/MS method has the advantages of qualitative and quantitative accuracy, it is fast and does not require analyte derivatization, and its sensitivity could allow for the detection of low-concentration substances. In recent years, LC–MS/MS has been commonly used to detect and analyze anticoccidial agents in animal-derived foods. Residues of anticoccidial drugs in food samples of animal origin usually require sample pretreatment steps, mainly including liquid–liquid extraction (LLE) [17,25]; solid-phase extraction (SPE) [18,31,33]; solvent front position extraction (SFPE) [20]; quick, easy, cheap, effective, rugged, and safe (QuEChERS) extraction [22,23]; gel permeation chromatography (GPC) [29]; and LLE–SPE [19,27]. The egg matrix is complex and rich in phospholipids, cholesterol, proteins, etc. Eggs usually need to undergo sample pretreatment to obtain a good extraction solution, thereby improving the recovery of the target compounds. According to the reported literature [18,31,33], egg samples are usually extracted via solid-phase extraction to improve the recovery of the target compounds. Solid-phase extraction is beneficial for enriching target compounds in samples while extracting and purifying them, thereby improving detection sensitivity.

The purpose of this study was to use LLE extraction and SPE purification and to detect robenidine (ROB), halofuginone (HAF), lasalocid (LAS), monensin (MON), nigericin (NIG), salinomycin (SAL), narasin (NAR), and maduramicin (MAD) residues in eggs by HPLC–MS/MS and UPLC–MS/MS. In this experiment, different sample preparation methods were optimized and compared, and the best extraction method was selected. Compared with previous detection methods, the UPLC–MS/MS method developed in this experiment has the advantages of a short run time, efficiency, and high recovery and precision. This research developed two efficient, rapid, and selective detection methods for the determination of eight anticoccidial drugs in eggs, providing a scientific basis for monitoring anticoccidial drugs in eggs.

## 2. Results and Discussion

### 2.1. Sample Clean-Up

**Figure 1:**
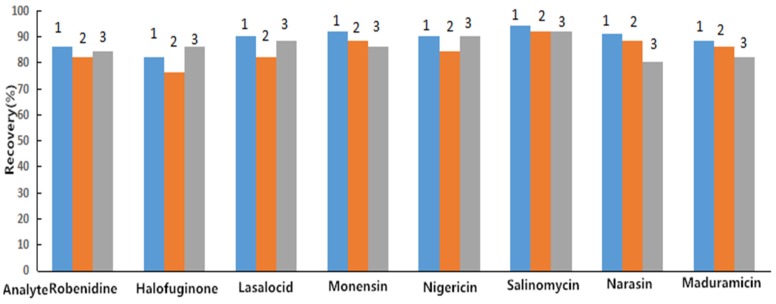
Comparison of the recovery of eight coccidiostats in eggs via different solid-phase extraction cartridges. Note: 1. CNW C_18_ cartridge; 2. SIMON C_18_ cartridge; 3. BOSHI C_18_ cartridge.

The sample preparation step is an indispensable step for extracting and purifying samples to prevent impurities from clogging the column or disrupting the liquid chromatographic separation. According to previous reports [17,19,25], LLE and LLE–SPE methods were used to extract and purify egg samples in this experiment. After extraction and purification by LLE, the recovery of eight anticoccidial drugs in eggs was determined in this study. The results showed that the target compounds had large interference peaks at low concentrations (1.0 ng/mL), and the recovery rates of ROB, HAF, and MAD were between 51.3% and 69.0%, which did not meet the verification requirements of the European Union (EU) and US Food and Drug Administration (FDA) recovery rates. Therefore, this study used LLE combined with SPE to extract and purify egg samples. Choosing a suitable SPE cartridge is one of the important factors that determines the sufficient extraction ability of SPE. In preliminary experiments, Strata-XC cartridges (3 mL/60 mg, Phenomenex Corp., USA), MCX cartridges (3 mL/60 mg, CNW Technologies GmbH., Germany), and PCX cartridges (6 mL/150 mg, CNW Technologies GmbH., Germany) were used to extract and purify egg samples. The results showed that the recovery rates of the eight anticoccidial drugs were all low. Considering that incomplete adsorption may have been occurring in the extraction cartridges, corresponding proportions of formic acid or acetic acid were added to the extraction solution and the equilibrium solution to adjust the pH values. Compared with previous results, the ROB and HAF recovery rates were relatively low, and the recovery rates of the other drugs met EU validation requirements. Optimization of the ratio of formic acid or acetic acid in the eluent was conducted, and we found that there was no significant difference between the addition of 5%, 10%, and 15% formic acid or acetic acid, which proved to be unrelated to the eluent. Based on our previous research [27], this study compared the effects of different C18 cartridges on extraction recovery at concentrations of 25.0 μg/kg ROB and HAF, 37.5 μg/kg MON, 60.0 μg/kg MAD, and 15.0 μg/kg LAS, NIG, SAL, and NAR, including CNW C18 cartridges (6 mL/150 mg, CNW Technologies GmbH., Germany); SIMON C18 cartridges (6 mL/150 mg, Simon Aldrich Bio & Chemicals Ltd., Germany); and BOSHI C18 cartridges (6 mL/500 mg, BoShi Biotechnology Co., Ltd., China). As shown in Figure 1, the three C18 cartridges achieved good extraction recoveries, and the CNW C18 cartridge (6 mL/150 mg, CNW Technologies GmbH., Germany) had better results than those of the other two cartridges. Thus, CNW C18 cartridges were used in this study to extract and purify the target compounds from eggs. In addition, an automated solid-phase extraction instrument was used in this study, and a program was set for automatic extraction according to our previous research [27]. In this experiment, liquid–liquid extraction combined with automated solid-phase extraction was used to extract and purify eight anticoccidial drugs in eggs with high recovery rates.

### 2.2. Selection of the Chromatographic Column and Mobile Phase

ROB and HAF have relatively high polarities, which are more likely to be eluted together with highly polar impurities in the matrix extract [27,31,33]. For complete separation and optimal retention of these two highly polar analytes on the chromatographic column, the selection and optimization of the chromatographic column, mobile phase, and gradient elution procedures were critical. 

The reported studies on the determination of these anticoccidials drugs mostly use C18 [17,18,19,20,23,24,25,26,27,28,29] or C8 [21,22,30] columns and involve separation from animal-derived foods. Most of the anticoccidial drug residues in eggs are separated by C18 columns because the carbon chain is long, which increases the area of nonpolar interaction of the bonded phase and affects retention and selectivity. Therefore, good separation can be achieved. In this experiment, a Waters SunFireTM C18 column (150 mm × 4.6 mm; inside diameter (i.d.) 5 μm) and a Waters ACQUITY UPLC BEH C18 column (50 mm × 2.1 mm; i.d. 1.7 μm) were used to separate eight coccidiostats in eggs via HPLC and UPLC, respectively. In our previous research [27], the mobile phase used in the gradient elution was composed of a 0.1% formic acid–water solution and pure methanol, which could sufficiently isolate eight coccidiostats from beef. When a 0.1% formic acid–water solution and pure methanol were used as the mobile phase, LC–MS/MS analysis revealed that the eight target compounds in eggs exhibited a tailing phenomenon. Due to the fact that the composition of eggs, especially the yolk, is more complex than the compositions of other matrices, this causes interference with these target compounds. To solve the problem of peak tailing for these target analytes, this study added 2, 5, 10, and 20 mM ammonium acetate to the 0.1% formic acid–water solution to improve the resolution and peak shape of the analytes. The experimental results showed that when 10 mM ammonium acetate was added to the 0.1% formic acid–water solution, the peak shape of these target analytes was significantly improved. Finally, the mobile phase composition utilized in this experiment was 10 mM ammonium acetate–0.1% formic acid in water (A) and methanol (B). Moreover, the experiment used a gradient elution procedure, and the ratio of phase A and phase B were optimized to obtain good peak shapes and retention times for the target analytes.

### 2.3. Optimization of Mass Spectrometric Conditions

A 1.0 μg/mL standard working solution of the eight anticoccidial drugs was prepared in methanol–0.1% formic acid aqueous solution (50:50, *v*/*v*) and utilized as the mass spectrometry tuning solution. A certain amount of formic acid was added to facilitate protonation of the analytes and to obtain a higher response value. The full-scan MS/MS spectra of ROB, HAF, LAS, MON, NIG, SAL, NAR, and MAD were obtained by infusing 1 μg/mL standard working solutions into the ESI(+) source. In MS mode, the sodium adducts [M + Na]^+^ were selected as the precursor ions for NIG and NAR at *m*/*z* 747.4 and 787.6, respectively, whereas the precursor ions for ROB, HAF, LAS, MON, SAL, and MAD were the protonated molecular ions [M + H]^+^ at m/z 333.9, 416.1, 613.3, 693.4, 773.5, and 934.5, respectively, which agreed with the descriptions of Olejnik et al. [33] and Clarke et al. [41]. In multiple reaction monitoring (MRM) mode, one precursor ion and two product ions were selected to assess the detection of the eight coccidiostats. The most abundant product ion was selected as the quantitative ion for the analyte, while the other ions were used to qualitatively analyze the target compound. As shown in Table 1, the *m*/*z* values of the quantification ions of ROB, HAF, LAS, MON, NIG, SAL, NAR, and MAD were 138.0, 100.1, 377.2, 461.3, 657.4, 431.3, 431.4, and 629.3, respectively. The declustering potential and collision energy values were optimized based on the monitored ion pairs of the eight coccidiostats, and MRM mode was used to detect the target compounds.

**Figure 2:**
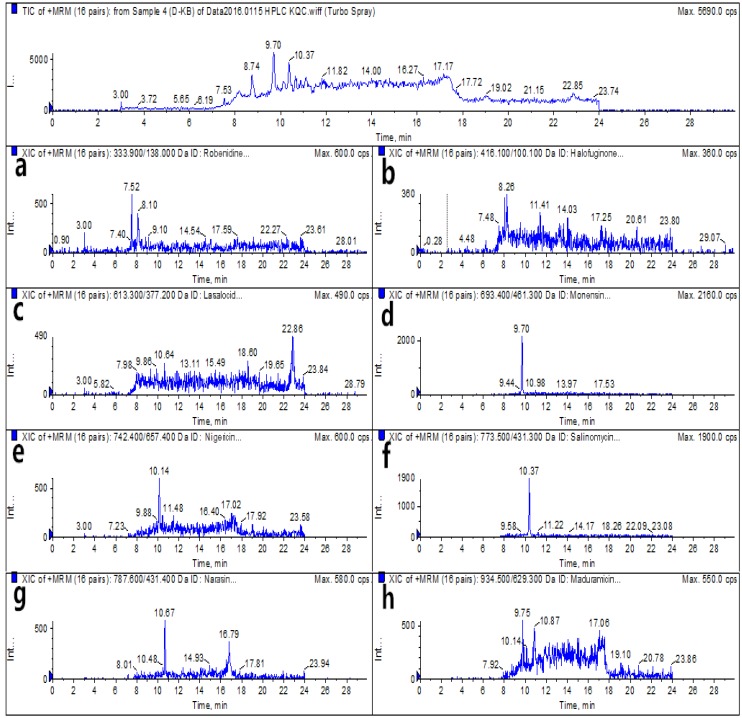
HPLC–MS/MS total ion chromatograms (TICs) and extracted ion chromatograms (XICs) (**a**–**h**: robenidine (ROB), halofuginone (HAF), lasalocid (LAS), monensin (MON), nigericin (NIG), salinomycin (SAL), narasin (NAR), and maduramicin (MAD)) of eight coccidiostats in a blank egg sample. Note: “Int ...” is represented as "Intensity, cps" in the figure, the following is the same.

**Figure 3:**
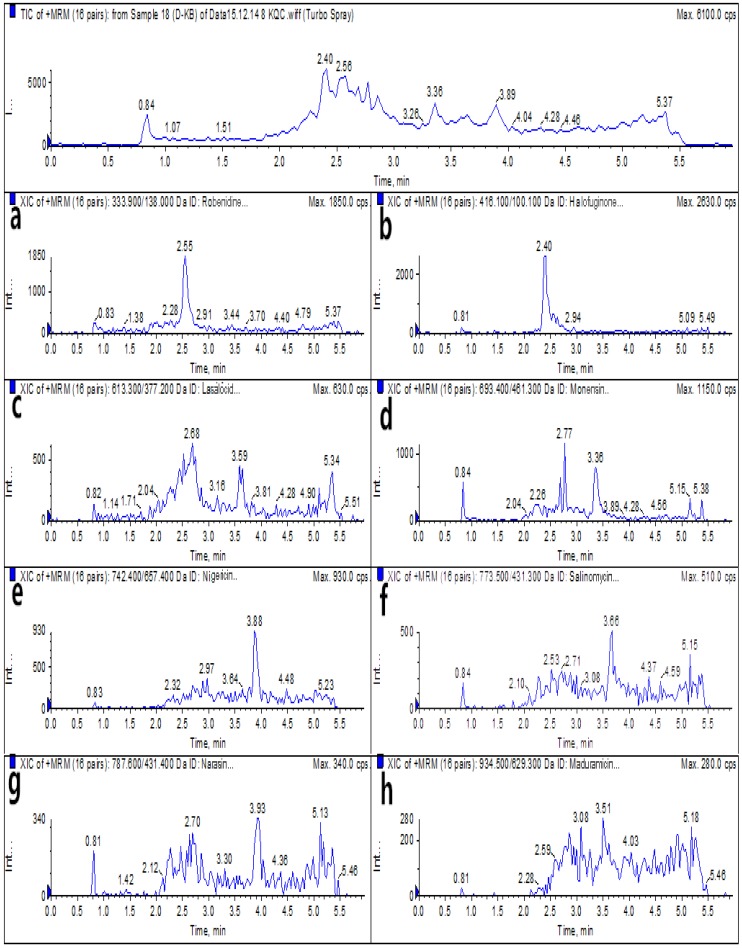
UPLC–MS/MS TICs and XICs (**a**–**h**: ROB, HAF, LAS, MON, NIG, SAL, NAR, and MAD) of eight coccidiostats in a blank egg sample.

**Figure 4:**
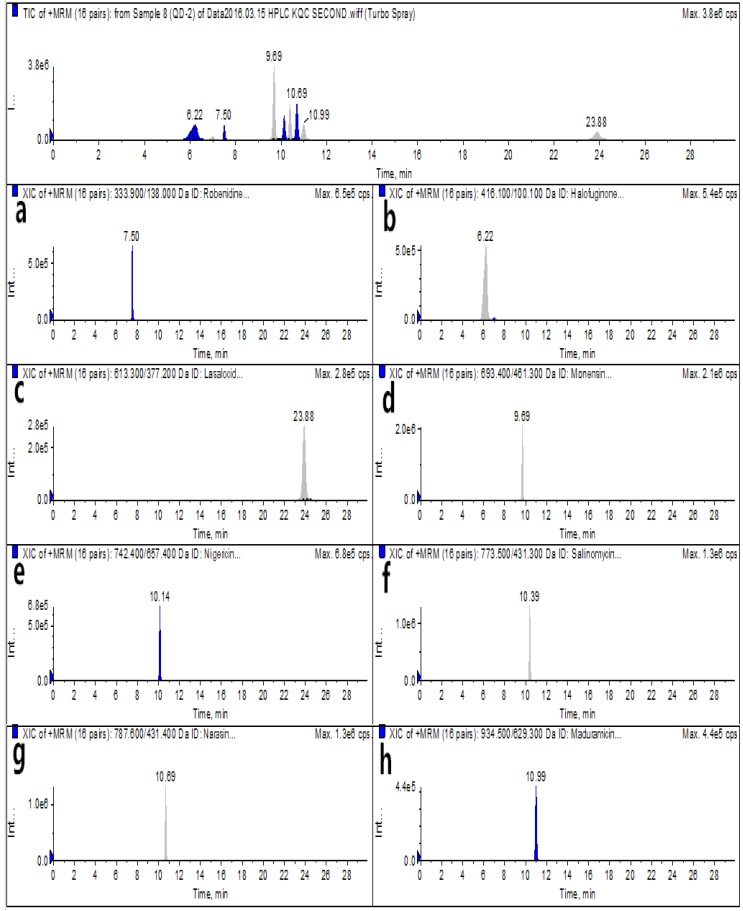
HPLC–MS/MS TICs and XICs (**a**–**h**: ROB, HAF, LAS, MON, NIG, SAL, NAR, and MAD) of a blank egg sample spiked with ROB and HAF at 25.0 μg/kg, MON at 37.5 μg/kg, MAD at 60.0 μg/kg, and LAS, NIG, SAL, and NAR at 15.0 μg/kg.

**Figure 5:**
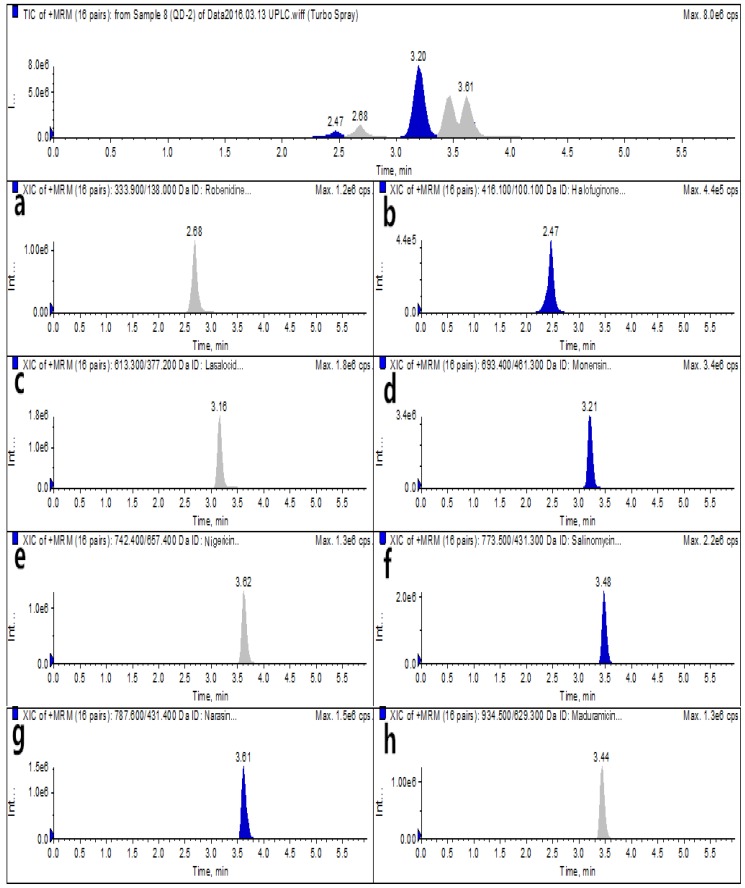
UPLC–MS/MS TICs and XICs (**a**–**h**: ROB, HAF, LAS, MON, NIG, SAL, NAR, and MAD) of a blank egg sample spiked with ROB and HAF at 25.0 μg/kg, MON at 37.5 μg/kg, MAD at 60.0 μg/kg, and LAS, NIG, SAL, and NAR at 15.0 μg/kg.

For analysis via the optimized HPLC–MS/MS and UPLC–MS/MS methods, blank egg samples and spiked samples were prepared before the authentic samples were subjected to detection by the instruments. The total ion chromatograms (TICs) and extracted ion chromatograms (XICs) of the eight coccidiostats are shown in Figure 2 and Figure 3 for blank egg samples. The TICs and XICs of the quantitative ions of blank egg samples spiked with 25.0 μg/kg ROB and HAF, 37.5 μg/kg MON, 60.0 μg/kg MAD, and 15.0 μg/kg LAS, NIG, SAL, and NAR are shown in Figure 4 and Figure 5. Compared to Figure 2 and Figure 3, Figure 4 and Figure 5 show that the eight coccidiostats have sharp peaks with no tailing, and the blank egg samples do not contain artefacts of these target analytes.

### 2.4. Bioanalytical Method Validation

The HPLC–MS/MS and UPLC–MS/MS methods were validated according to European Union Regulation 2002/657/EC [42], as well as according to certain important parameters from the FDA [43], whereas the specificity of the two methods was tested by analyzing blank egg samples. The two detection methods showed good linearity within the analyzed ranges of the target analytes, and the correlation coefficients (R^2^ values) were all greater than 0.999 51, as listed in Table 2.

Residues of these anticoccidial drugs in eggs can seriously harm consumers’ health. The EU banned the use of these anticoccidial drugs in poultry laying, but illegal farms still use them. However, the European Union [42] did not set a maximum residue limit (MRL) for these drugs. This experiment studied the recovery and precision of the eight coccidiostats based on their MRLs in chicken muscle established in China [44]. A total of 2.0 g of blank egg sample was spiked with eight coccidiostats at four concentrations (the LOQ, 0.5 MRL, 1 MRL, and 2 MRL), and after sample pretreatment the corresponding concentrations in the matrix extracts were obtained. Recovery and precision were evaluated by measuring the extracts at these four concentrations, and each concentration was measured six times. As shown in Table 3, the recoveries of the eight coccidiostats in the blank egg samples were 71.69%–96.52%, the relative standard deviations (RSDs) were 2.64%–15.34%, the intraday RSDs were 2.10%–18.72%, and the interday RSDs were 3.61%–18.47% for HPLC–MS/MS. As shown in Table 4, the recoveries of eight coccidiostats in the blank egg samples were 72.26%–102.74%, the RSDs were 3.69%–13.25%, and the intraday RSDs and the interday RSDs were 6.72%–12.28% and 8.15%–15.42%, respectively, for UPLC–MS/MS.

According to the EU methodological parameter requirements [42], recovery rates greater than or equal to 70%, and RSD values less than or equal to 20% are sufficient. As shown in Table 3 and Table 4, the recovery and precision of the two methods meet the EU method parameter verification requirements.

The LOQ and LOD values were determined for the HPLC–MS/MS and UPLC–MS/MS methods and are shown in Table 2. The matrix effect formula was matrix effect (ME) = (A − B)/B × 100%, where A is the average peak area of the matrix standard and B is the average peak area of the solvent standard. According to the formula, the matrix effects of the eight anticoccidial drugs in eggs were determined for the UPLC–MS/MS methods at concentrations of 0.5 MRL, 1.0 MRL, and 2.0 MRL. As shown in Table 5, ROB and HAF showed matrix enhancement effects, while the other six polyether drugs showed matrix inhibition effects. In this study, by optimizing the chromatographic conditions and mobile phases, the matrix effects were reduced, and the recovery and precision of the method for the target analytes were improved.

### 2.5. Comparison with Other LC–MS/MS Methods

Most researchers use HPLC–MS/MS and UPLC–MS/MS methods to detect anticoccidial drug residues in eggs [17,18,19,25,29,31,33]. The developed method and other previously reported methods for the determination of anticoccidial drug residues in egg samples were compared based on different parameters (LOD, LOQ, recovery, RSD, and run time) in Table 6. Spisso et al. [25] reported an LLE–HPLC–MS/MS method for the simultaneous determination of LAS, MON, SAL, NAR, and MAD in eggs. The sample run time of this method was 18 min, and the precision was between 4.4% and 15.7%. Chico et al. [29] established a GPC–UPLC–MS/MS method to detect LAS, MON, SAL, NAR, and MAD in eggs, and the recovery and precision of the target compounds were 55.3–77.5% and 4.2–15.6%, respectively, and the sample run time was 11 min. Galarini et al. [31] developed an SPE–HPLC–MS/MS method for the qualitative analysis of seven coccidiostats in eggs, and the recovery and precision of these target compounds were 89.0–113.0% and 3.4–16.0%, respectively, and the run time was 40 min. In this study, LLE–SPE was used for sample pretreatment, and eight coccidiostats in eggs were detected by HPLC–MS/MS and UPLC–MS/MS. The recovery and precision of these two methods were higher than those of the previously reported methods, and the run time was greatly reduced. Compared with those of the LLE and GPC methods, the recovery rates of the eight coccidiostats in eggs extracted by LLE–SPE were higher. Compared with the HPLC–MS/MS method, the UPLC–MS/MS method has the advantages of less reagent consumption, high recovery and precision, and a shorter detection time. Thus, this study established LLE–SPE–HPLC–MS/MS and LLE–SPE–UPLC–MS/MS methods for the simultaneous determination of eight coccidiostats in eggs and provided new techniques and a scientific basis for the detection of eight coccidiostat residues in eggs.

### 2.6. Real Sample Analysis

To evaluate the applicability of the method, we purchased 40 eggs from a local farm for analysis by HPLC–MS/MS and UPLC–MS/MS. The 40 eggs were extracted by liquid–liquid extraction, purified by solid-phase extraction, and analyzed on the instruments. It was found that only one egg contained 4.57 μg/kg and 4.86 μg/kg MON residues detected by HPLC–MS/MS and UPLC–MS/MS, respectively, but the eggs did not contain any of the other drugs. Therefore, the optimized LLE–SPE–HPLC–MS/MS and LLE–SPE–UPLC–MS/MS methods can be applied to quantify eight coccidiostats in egg samples.

## 3. Materials and Methods

### 3.1. Chemicals and Reagents

Robenidine (95.0% standard) and lasalocid (94.0% standard) were obtained from LGC Labor GmbH (Augsburg, Germany). Halofuginone (98.0% standard) and narasin (95.0% standard) were purchased from Toronto Research Chemicals Inc. (Toronto, Canada). Monensin (95.0% standard), salinomycin (79.8% standard), and maduramicin (96.0% standard) were purchased from Labor Dr. Ehrenstorfer-Schafers (Augsburg, Germany). Nigericin (99.0% standard) was obtained from EMD Millipore Corp. (Billerica, MA, USA). Acetonitrile and methanol (HPLC grade) were purchased from Merck (Kenilworth, NJ, USA). Hexane (Analytical-grade) was acquired from Sinopharm Chemical Reagent Co. (Shanghai, China). Ultrapure water was obtained from a PURELAB Option-Q synthesis water purification system (ELGA Lab Waters, High Wycombe, Bucks, UK).

### 3.2. Preparation of the Standard Stock and Working Solutions

Standard stock solutions of the eight coccidiostats were prepared at 1.00 mg/mL in pure methanol. A mixed standard stock solution of the eight coccidiostats (100.0 μg/mL) was prepared by adding 0.1 mL of each target compound standard stock solution (1.00 mg/mL) to 10 mL of pure methanol. The individual standard working solutions and mixed standard working solutions were prepared by diluting the above-mentioned individual standard stock solutions and the mixed standard stock solution with pure methanol, respectively, the respective concentrations of which were formulated according to the linearity and recovery parameters. The standard stock solutions and working solutions were stored at −75 °C and −34 °C, respectively.

### 3.3. LC–MS/MS Instrumentation and Conditions

#### 3.3.1. HPLC Method

HPLC analysis was performed using a Waters Alliance e2695 separation module (Waters Corp., Milford Massachusetts, USA), and separations were achieved using a Waters SunFireTM C18 column (150 mm × 4.6 mm; i.d. 5 μm), protected with a guard column (Waters XBridgeTM C18; 20 mm × 4.6 mm; i.d. 5 μm), and maintained at 50 °C. The mobile phase used in the elution gradient was composed of 0.1% formic acid in water (A) and methanol (B), and A was mixed with 10 mM ammonium acetate. The flow rate was 0.8 mL/min, and the injection volume was 10 μL. The gradient started at 60% A for 4 min, which was then decreased to 0% in 1 min and maintained for 9 min, increased to 5% in 1 min and maintained for 10 min, and, finally, increased to 60% in 1 min and maintained for 4 min.

#### 3.3.2. UPLC Method

UPLC analysis was performed using a Waters ACQUITY UPLCTM system (Waters Corp., Milford, Massachusetts, USA), and separation was achieved using a Waters ACQUITY UPLC BEH C18 column (50 mm × 2.1 mm; i.d. 1.7 μm), protected with a guard column (Waters ACQUITY UPLC BEH C18; 5 mm × 2.1 mm; i.d. 1.7 μm), and maintained at 30 °C. The injection volume was 10 μL. A gradient elution was performed with solutions A (10 mM ammonium acetate–0.1% formic acid in water) and B (methanol) at 0.3 mL/min: 0–1 min, 96% A; 2–4.5 min, 10% A; and 5–6 min, 96% A.

#### 3.3.3. Mass Spectrometric Conditions

A triple quadrupole mass spectrometer (AB SCIEX Triple QuadTM 5500, AB SCIEX Corp., Framingham, Massachusetts, USA) was used to qualitatively and quantitatively analyze the target compounds, and Analyst version 1.6.1 software (AB SCIEX Pte. Ltd., Concord, Ontario, Canada) was used for the analysis. Tandem mass spectrometry was used to determine the precursor and product ions of the target compounds in positive ionization and multiple reaction detection (MRM) modes. The typical MS parameters were as follows: electrospray ionization voltage, 5500 V; ion source temperature, 550 °C; spray gas, auxiliary gas (nitrogen), curtain gas, and collision gas pressure, 45, 50, 35, and 8 psi, respectively; collision chamber outlet voltage, 12 V; and injection voltage, 10 V. The precursor ions, product ions, the corresponding cone voltage, and collision energy of the eight coccidiostats are listed in Table 1.

### 3.4. Sample Extraction and Purification

#### 3.4.1. Liquid–Liquid Extraction

Homogeneous blank egg samples (2.0 ± 0.02 g) were accurately weighed into 50 mL centrifuge tubes, and 10 mL acetonitrile was added. After vortexing for 2 min and ultrasonication for 10 min, the mixture was centrifuged at 5000× *g* and 4 °C for 5 min. The supernatant was collected into another 50 mL centrifuge tube. The sample was extracted again, and the extracted supernatant was collected twice. Five milliliters of an acetonitrile-saturated n-hexane solution was added to the extract, vortexed for 1 min, and then centrifuged at 5000× *g* and 25 °C for 5 min. The upper n-hexane layer was discarded, and the extraction was repeated once more. 

#### 3.4.2. Liquid–Liquid Extraction and Solid-Phase Extraction

Homogeneous blank egg samples (2.0 ± 0.02 g) were accurately weighed into 50 mL centrifuge tubes. After repeating the above liquid–liquid extraction steps, the extract was subjected to solid-phase extraction. The steps of solid-phase extraction were as follows: Strata-X-C cartridges (3 mL/60 mg, Phenomenex Corp., Torrance, CA, USA), MCX cartridges (3 mL/60 mg, CNW Technologies GmbH., Düsseldorf, Germany), and PCX cartridges (6 mL/150 mg, CNW Technologies GmbH., Germany) were activated and equilibrated with the addition of 5 mL of methanol and 5 mL of ultrapure water. After 25 mL of the extract was added and dried at a constant rate, 3 mL of ultrapure water and 3 mL of methanol were added to the cartridge, and then 3 mL of ammonium hydroxide was added to elute the target compounds.

#### 3.4.3. Liquid–Liquid Extraction and Automated Solid-Phase Extraction

Homogeneous blank egg samples (2.0 ± 0.02 g) were accurately weighed into 50 mL centrifuge tubes, and 2 mL of ultrapure water and 8 mL of (acetonitrile: ethyl acetate = 60: 40, v/v):acetic acid (98:2, v/v) were added. After vortexing for 1 min and ultrasonication for 10 min, the mixture was centrifuged at 5000 ×g and 4 °C for 5 min. The supernatant was collected into another 50 mL centrifuge tube. The sample was extracted again, and the extracted supernatant was collected twice and then evaporated to dryness under a nitrogen stream in an N-EVAP 111 nitrogen evaporator (Organomation Associates Inc., Berlin, MA, USA) at 40 °C. Five milliliters of acetonitrile was added to redissolve the dried sample, and 1 mL of an acetonitrile-saturated n-hexane solution was used to remove any fats. The defatting step was repeated once more, and 20 mL of ultrapure water was used to dilute the sample. The steps of automatic solid-phase extraction (SPE-10, Bona Agere Technology Co., Ltd., Tianjin, China) were as follows: C18 cartridges (6 mL/150 mg, CNW Technologies GmbH., Germany) were activated and equilibrated with the addition of 5 mL of methanol and 5 mL of ultrapure water, and the flow rate was 2 mL/min. After the extract was added and dried at 2 mL/min, 6 mL of ultrapure water and 3 mL of 5% methanol was added to the cartridges at 3 mL/min, and then 15 mL of ethyl acetate was added to elute the target compounds at 1 mL/min.

#### 3.4.4. Sample Purification

After the target analytes were eluted, the eluate was dried under a nitrogen stream at 40 °C. The residue was redissolved in 2 mL of methanol, transferred to a 2 mL centrifuge tube, and centrifuged at 14 000 ×g for 10 min. Afterwards, the extract was syringe filtered (0.22 μm filter), and 10 μL of the extract was injected into the LC–MS/MS system.

### 3.5. Method Validation

The linearity of this method was evaluated by establishing calibration curves, which were generated by spiking blank egg samples with the eight coccidiostats. Matrix-matched calibration curves were prepared at nine spiking levels (LOQ, 2.0, 5.0, 10.0, 20.0, 40.0, 60.0, 80.0, and 100.0 μg/kg) for each coccidiostat, which were constructed using the analyte peak area versus the analyte concentration.

When the signal-to-noise (S/N) ratio of the eight coccidiostats in the egg sample matrix solution was determined to be 3 and 10, the corresponding spiked concentrations were used as the LODs and LOQs of the target compounds in the egg sample matrix, respectively. The corresponding recovery, precision, and matrix effects values were established according the EU and the FDA [42,43].

## 4. Conclusions

In this study, we developed a sample preparation method of LLE combined with SPE to extract and purify eight coccidiostat residues in eggs. The LLE–SPE method can provide good extraction recoveries of the target analytes in eggs. These HPLC–MS/MS and UPLC–MS/MS methods for the quantitative determination of eight coccidiostats are accurate and sensitive. Moreover, compared with the merits of the HPLC–MS/MS method, the UPLC–MS/MS method was faster and had higher recovery and precision values. These two methods meet the requirements of the Chinese Ministry of Agriculture, the EU, and the FDA for detecting eight coccidiostats in eggs and were successfully applied for the analysis of 40 egg samples, proving the feasibility of these two methods.

## Figures and Tables

**Table 1:** Mass spectral parameters, precursor ions, and product ions of eight coccidiostats.

Analyte	Precursor Ions (*m/z*)	Product Ions (*m/z*)	Declustering Potential (V)	Collision Energy (eV)
ROB	333.9	138.0* 155.0	59.1 59.1	47.0 47.0
HAF	416.1	100.1* 138.2	46.2 62.2	25.6 25.2
LAS	613.3	377.2* 577.4	66.0 56.9	48.4 44.0
MON	693.4	461.3* 479.3	68.0 68.0	68.3 67.8
NIG	747.4	657.4* 675.4	86.0 86.9	37.0 32.0
SAL	773.5	431.3* 531.4	59.1 59.1	65.6 54.5
NAR	787.6	431.4* 531.4	84.8 87.8	69.9 60.3
MAD	934.5	629.3* 647.4	24.8 24.8	31.1 27.9

Note: * Ion used for quantification.

**Table 2:** Linearity, determination coefficient, LODs, and LOQs for the analysis of eight coccidiostats in egg samples.

Method	Analyte	Linearity	Determination Coefficient (R^2^)	Linearity Range (μg/kg)	LOD (μg/kg)	LOQ (μg/kg)
HPLC–MS/MS	ROB	y = 34616x − 506.76	0.99983	0.82~100	0.23	0.82
	HAF	y = 61224x + 6543.7	0.99986	1.04~100	0.32	1.04
	LAS	y = 113825.898x − 45626	0.99951	1.46~100	0.44	1.46
	MON	y = 52901x + 14151	0.99995	1.21~100	0.37	1.21
	NIG	y = 67809x − 22104	0.99966	1.03~100	0.31	1.03
	SAL	y = 114639.404x + 26519	0.99974	1.68~100	0.51	1.68
	NAR	y = 107772.7495x + 20585	0.99986	1.45~100	0.44	1.45
	MAD	Y = 178339.3611x − 107324.0928	0.99984	1.73~100	0.52	1.73
UPLC–MS/MS	ROB	y = 292553.0261x + 16571	0.99993	0.81~100	0.24	0.81
	HAF	y = 100732.4827x − 769.94	0.99994	1.04~100	0.32	1.04
	LAS	y = 538339.9425x + 32878	0.99994	1.25~100	0.42	1.25
	MON	y = 463092.5586x + 30618	0.99993	1.08~100	0.18	1.08
	NIG	y = 593130.8977x− 29292	0.99976	0.87~100	0.16	0.87
	SAL	y = 719531.8524x − 96792	0.99979	0.85~100	0.19	0.85
	NAR	y = 715834.9244x − 129389.5518	0.99977	1.02~100	0.28	1.02
	MAD	y = 19688x + 2081	0.99976	0.99~100	0.22	0.99

**Table 3:** Recoveries and precision of eight coccidiostats spiked in blank eggs in HPLC–MS/MS analysis.

Analyte	Spiked Concentration (μg/kg)	Recovery (%) (*n* = 6)	RSD (%) (*n* = 6)	Intraday RSD (%) (*n* = 6)	Interday RSD (%) (*n* = 18)
ROB	0.82	76.28 ± 10.31	13.52	16.32	14.38
	50.00	85.42 ± 6.20	7.26	6.55	5.81
	100.00 ^a^	82.66 ± 6.89	8.34	6.47	6.58
	200.00	86.31 ± 5.39	6.24	7.23	8.02
HAF	1.04	74.58 ± 7.82	10.49	12.18	13.09
	50.00	86.44 ± 5.17	5.98	8.34	8.56
	100.00 ^a^	85.21 ± 4.53	5.32	6.17	5.89
	200.00	82.45 ± 5.47	6.63	5.84	4.35
LAS	1.46	76.25 ± 8.59	11.27	11.68	14.52
	300.00	83.61 ± 3.39	4.05	2.10	6.87
	600.00 ^a^	86.34 ± 2.28	2.64	5.58	7.65
	1200.00	85.20 ± 2.54	2.98	5.96	3.61
MON	1.21	76.98 ± 9.03	11.73	14.25	13.13
	750.00	86.77 ± 4.27	4.92	6.58	7.36
	1500.00 ^a^	94.35 ± 4.57	4.84	5.38	8.89
	3000.00	96.52 ± 4.92	5.10	5.98	6.74
NIG	1.03	76.65 ± 8.40	10.96	12.43	15.09
	300.00	81.26 ± 3.32	4.09	9.84	3.46
	600.00 ^a^	89.34 ± 4.18	4.68	5.86	7.82
	1200.00	89.98 ± 4.28	4.76	6.62	8.84
SAL	1.68	72.81 ± 11.17	15.34	16.25	15.43
	300.00	80.27 ± 4.33	5.39	5.49	8.46
	600.00 ^a^	89.80 ± 5.71	6.36	6.71	3.69
	1200.00	89.78 ± 4.75	5.29	3.56	8.55
NAR	1.45	71.69 ± 7.79	10.87	18.72	18.47
	300.00	84.57 ± 3.79	4.48	7.13	7.41
	600.00 ^a^	86.88 ± 3.27	3.76	6.98	6.83
	1200.00	86.50 ± 4.50	5.20	8.28	9.53
MAD	1.73	79.63 ± 7.88	9.90	9.68	11.34
	120.00	83.81 ± 4.79	5.72	8.34	7.62
	240.00 ^a^	86.62 ± 4.40	5.08	8.87	7.58
	480.00	84.37 ± 4.74	5.62	8.86	8.51

Note: a. MRLs.

**Table 4:** Recoveries and precision of eight coccidiostats spiked in blank eggs in UPLC–MS/MS analysis.

Analyte	Spiked Concentration (μg/kg)	Recovery (%) (*n* = 6)	RSD (%) (*n* = 6)	Intraday RSD (%) (*n* = 6)	Interday RSD (%) (*n* = 18)
ROB	0.81	81.23 ± 5.47	6.73	8.29	9.37
	50.00	86.68 ± 5.12	5.91	7.29	11.83
	100.00 ^a^	86.71 ± 5.32	6.14	9.01	11.07
	200.00	86.22 ± 5.22	6.05	7.20	11.10
HAF	1.04	72.26 ± 5.73	7.93	7.24	8.15
	50.00	77.57 ± 4.20	5.41	7.34	8.70
	100.00 ^a^	80.26 ± 4.38	5.46	7.13	9.19
	200.00	79.50 ± 2.93	3.69	7.75	8.63
LAS	1.25	75.64 ± 7.30	9.65	12.28	15.42
	300.00	82.75 ± 5.25	6.34	8.40	9.26
	600.00 ^a^	83.73 ± 4.18	4.99	8.36	10.46
	1200.00	82.35 ± 5.82	7.07	8.75	10.79
MON	1.08	80.36 ± 7.78	9.68	10.37	14.58
	750.00	90.69 ± 7.00	7.72	7.96	8.25
	1500.00 ^a^	90.44 ± 7.91	8.75	9.84	9.86
	3000.00	93.55 ± 6.93	7.41	8.51	8.88
NIG	0.87	81.39 ± 10.78	13.25	11.58	14.26
	300.00	99.52 ± 7.98	8.02	8.66	10.19
	600.00 ^a^	99.14 ± 8.60	8.67	9.14	9.55
	1200.00	93.74 ± 7.33	7.82	9.77	9.58
SAL	0.85	87.35 ± 8.16	9.34	9.48	11.25
	300.00	102.74 ± 4.95	4.82	7.62	9.84
	600.00 ^a^	102.04 ± 5.60	5.49	8.06	9.93
	1200.00	96.53 ± 5.89	6.10	8.55	9.99
NAR	1.02	74.93 ± 4.26	5.69	6.72	10.54
	300.00	88.51 ± 6.69	7.56	8.79	10.14
	600.00 ^a^	88.53 ± 6.24	7.05	7.22	8.42
	1200.00	86.14 ± 7.42	8.61	8.62	9.15
MAD	0.99	72.54 ± 7.44	10.26	9.38	11.76
	120.00	84.00 ± 4.80	5.71	7.97	9.86
	240.00 ^a^	82.42 ± 4.09	4.96	9.00	8.45
	480.00	83.93 ± 6.75	8.04	9.72	10.07

Note: a. MRLs.

**Table 5:** Matrix effects of the egg samples on eight coccidiostats at three levels in UPLC–MS/MS detection.

Added Levels (μg/kg)	Analyte	ROB	HAF	LAS	MON	NIG	SAL	NAR	MAD
0.5 MRL	Matrix effect (%)	15	16	−14	−31	−37	−41	−38	−51
1 MRL		19	24	−21	−34	−30	−37	−40	−55
2 MRL		22	18	−29	−33	−41	−38	−34	−52

**Table 6:** Comparison of the presented two methods with other methods utilized for egg analysis.

Detection Method	Sample Preparation Method	Analyte	LOD (μg/kg)	LOQ (μg/kg)	Recovery (%)	RSD (%)	Detection Time (min)	Ref.
HPLC–MS/MS	LLE	LAS, MON, SAL, NAR	1.0	1.2–1.6	90.3–112.7	5.4–13.9	14	Mortier et al. [17]
HPLC–MS/MS	SPE	LAS, MON, SAL, NAR	0.8–1.4	0.9–2.0	64.0–99.0	–	12	Rokka and Peltonen [18]
HPLC–MS/MS	LLE–SPE	ROB	–	5.0	62.0–76.0	7.1–13.7	11	Buiarelli et al. [19]
HPLC–MS/MS	LLE	LAS, MON, SAL, NAR, MAD	0.04–1.0	0.16–3.4	-	4.4–15.7	18	Spisso et al. [25]
UPLC–MS/MS	GPC	LAS, MON, SAL, NAR, MAD	-	-	55.3–77.5	4.2–15.6	11	Chico et al. [29]
HPLC–MS/MS	SPE	ROB, HAF, LAS, MON, SAL, NAR, MAD	-	-	89.0–113.0	3.4–16.0	40	Galarini et al. [31]
HPLC–MS/MS	SPE	ROB, HAF, LAS, MON, NIG, SAL, NAR, MAD	0.26–2.62	0.75–7.54	89.1–111.7	3.6–19.0	22	Olejnik et al. [33]
HPLC–MS/MS	LLE–SPE	ROB, HAF, LAS, MON, NIG, SAL, NAR, MAD	0.23–0.52	0.82–1.73	71.7–96.5	3.6–15.3	30	this method
UPLC–MS/MS	LLE–SPE	ROB, HAF, LAS, MON, NIG, SAL, NAR, MAD	0.16–0.42	0.81–1.25	72.3–102.7	3.7–13.3	6	this method

Note: “-” Not reported.
